# ADAM Proteases in Cancer: Biological Roles, Therapeutic Challenges, and Emerging Opportunities

**DOI:** 10.3390/cancers17101703

**Published:** 2025-05-19

**Authors:** Sakshi Arora, Andrew M. Scott, Peter W. Janes

**Affiliations:** 1 Tumour Targeting Laboratory, Olivia Newton-John Cancer Research Institute, Melbourne, VIC 3084, Australia; 2 School of Cancer Medicine, Latrobe University, Melbourne, VIC 3083, Australia

**Keywords:** ADAM proteases, cancer progression, metalloproteinase inhibitors, therapeutic resistance, iRhoms, tetraspanins, proteolytic shedding, antibody–drug conjugates

## Abstract

ADAM (A Disintegrin and Metalloproteinase) family members are multifunctional transmembrane proteases that govern tumorigenesis and metastasis by cleaving membrane-bound substrates such as growth factors, cytokines, and cell adhesion molecules. Several ADAMs, including ADAM8, ADAM9, ADAM10, ADAM12, and ADAM17, are overexpressed in malignancies and are linked with a poor prognosis. These proteases contribute to tumour growth by regulating cell proliferation, cell fate, invasion, angiogenesis, and immune evasion. ADAM10 and ADAM17, especially, facilitate the shedding of critical developmental and growth factors and their receptors, as well as immuno-regulatory molecules, hence promoting tumour progression, immune escape, and resistance to therapy. Recent work has unveiled multiple regulatory pathways that modulate ADAM functions, which include trafficking, dimerization, and conformational modifications that affect substrate accessibility. These observations have rekindled efforts to produce selective ADAM inhibitors, avoiding the off-target consequences reported with early small molecule inhibitors targeting the enzyme active site, which is conserved also in matrix metalloproteinases (MMPs). Promising approaches tested in preclinical models and, in some cases, clinical settings include more selective small-molecule inhibitors, monoclonal antibodies, and antibody–drug conjugates designed to specifically target ADAMs. In this review, we will discuss the emerging roles of ADAMs in cancer biology, as well as the molecular processes that control their function. We further discuss the therapeutic potential of targeting ADAMs, with a focus on recent advances and future directions in the development of ADAM-specific cancer therapies.

## Simple Summary

Cancer remains a leading cause of death worldwide, with metastasis accounting for the majority of fatalities. A family of proteins called ADAM metalloproteases plays important roles in cancer progression by enabling tumour cells to grow, spread, and evade the immune system. These proteins help activate growth factors and other molecules that allow tumours to survive and resist treatment. Although previous attempts to block related enzymes faced challenges, new research has uncovered potential ways to target ADAMs without affecting healthy tissues. This review highlights how ADAM proteins contribute to cancer and discusses the latest advances in developing therapies to block their activity. Understanding and selectively targeting these proteins could lead to safer and more effective treatments for cancer patients.

## 1. Introduction

Cancer is a significant worldwide health concern and a leading cause of death, impacting the average lifespan in all nations [1]. Metastasis, the dissemination of cancer cells to distant organ sites, accounts for the majority of cancer-related fatalities, exceeding 90% [2]. Extracellular proteases are critical modulators of cell–cell and cell–extracellular matrix (ECM) interactions, which are fundamental to tumorigenesis and metastatic processes, making them longstanding targets for anticancer drug development [3]. Matrix metalloproteinases (MMPs), a class of zinc-dependent endopeptidases, are key facilitators of ECM degradation and cancer progression. Despite extensive research, MMP-targeted therapies have not achieved clinical success [4,5,6]. ADAMs (A Disintegrin and Metalloproteinase), which are also zinc-dependent and structurally related to MMPs, play crucial roles in mediating cellular signalling. Approximately half of ADAMs exhibit proteolytic activity. These are predominantly transmembrane proteins that regulate the shedding of the ectodomain of a wide variety of substrates, including membrane-bound cytokines and growth factors and their receptors, as well as cell adhesion molecules, influencing multiple aspects of tumorigenesis [3,5,7,8]. ADAMs are generated in the endoplasmic reticulum and develop in the Golgi region prior to being transported to the cell membrane [9]. Invertebrates and vertebrates share 38 members of the ADAM family, 13 of which are proteolytically functional (ADAMs 8, 9, 10, 12, 15, 17, 19, 20, 21, 28, and 30) [10,11].

## 2. Structure of ADAMs

**Figure 1:**
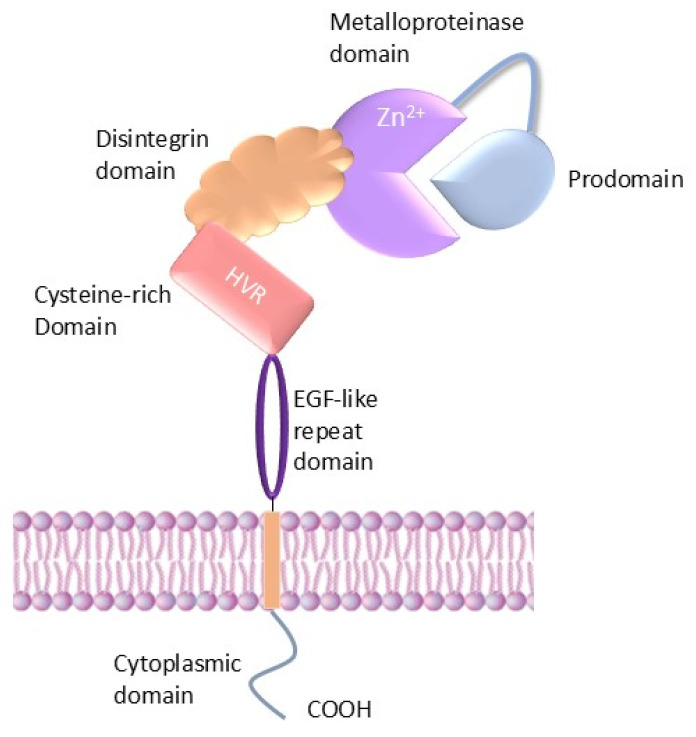
Structure of ADAMs. The ADAM extracellular region is composed of a prodomain, a metalloprotease domain, a disintegrin domain, a cysteine-rich domain with a hypervariable region (HVR), and usually an EGF-like domain; this is followed by the transmembrane and cytoplasmic domains. The Zn^2+^ binding site of the metalloprotease domain is substantially conserved throughout the metalloprotease family. Created with BioRender.com.

ADAMs have a conserved multi-domain structure consisting of an extracellular domain that comprises a prodomain with two convertase cleavage recognition sites, a Zn^2+^-dependent metalloprotease catalytic domain, a disintegrin domain, a cysteine-rich domain with a hypervariable region (HVR), an EGF-like repeat domain (except in ADAM10 or 17), a transmembrane domain, and a carboxy-terminal cytoplasmic domain (Figure 1).

The prodomain functions as a molecular chaperone and inhibits zymogen activity [12]. It also works in conjunction with the signal sequence (SS) to guide the intracellular transport of the immature protein [13]. Furin cleavage of the prodomain enhances the proteolytic activity, although it is not universally required, as ADAM8 can undergo autocatalytic prodomain removal within the trans-Golgi network [14,15,16]. This self-activation mechanism requires the multimerization of the ADAM8 precursor and the autocatalytic cleavage of its prodomain [17].

Catalytic action is provided by the metalloprotease (MP) domain, which has a conserved HEXGH sequence in common with MMPs. Proteolytic ability is lost in some ADAMs such as ADAM11, ADAM22, and ADAM23 due to mutations at this region, where the primary function of these subfamily members is thought to be in regulation of cell–cell/cell–matrix adhesion [16].

Towards the C-terminus from the metalloprotease domain lies in the disintegrin (D) domain, which can attach to integrin molecules and facilitate connections between cells and between cells and their matrices [18]. Following on from this is the cysteine-rich (C) domain, which, at least in ADAM10, forms a continuous structure with the disintegrin domain [19,20]. The C domain is important for the regulation of protease activity and substrate identification and binding. It also modulates integrin interaction with the disintegrin domain [21]. Following the C domain, most ADAMs, apart from the evolutionary distinct ADAMs 10 and 17, have a 30–40-amino-acid epidermal growth factor (EGF)-like domain, the function of which is unknown.

A transmembrane (TM) domain stabilizes the adjoining and intrinsically unstructured cytoplasmic region, and for ADAM10, it has been suggested to promote homodimerization in the membrane [22], while for ADAM17, it facilitates interaction with the cofactor protein iRhom2 to regulate trafficking and maturation (see below). The TM domain likely also supports other protein and lipid interactions of ADAMs via association with distinct lipid microdomains [22,23,24].

Finally, the cytoplasmic or C-terminal domain of the ADAM family members consists of an SH3-binding site [25] and varies in length and sequence, and its phosphorylation can modulate shedding activity, exemplified by threonine phosphorylation of ADAM17 [26]. However, not all ADAM proteins possess strong SH3 domain-binding capability, since an SH3 domain phage display screen [27] found that only ADAM8, ADAM9, and ADAM15 showed a strong and selective recovery of SH3-binding phages, indicating high-affinity and specific interactions [27]. Interestingly, at least for ADAMs 10 and 17, the cytoplasmic domain is not required for shedding activity, thus arguing for the existence of additional regulatory mechanisms [15].

## 3. Mechanisms Regulating ADAM Metalloproteases

**Figure 2:**
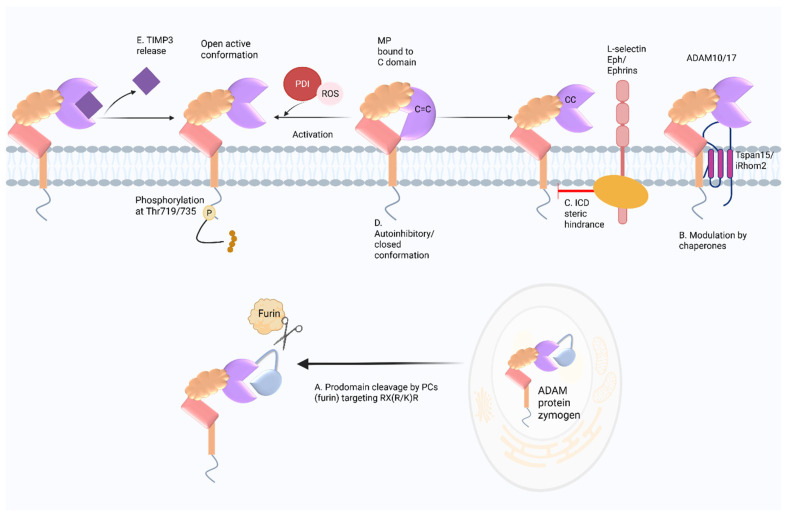
Various models for the modes of ADAM regulation. (**A**) Prodomain cleavage and activation: ADAM proteins are synthesized as zymogens and are retained in the endoplasmic reticulum (ER) until they are transported through the secretory pathway. Proprotein convertases such as furin cleave the prodomain at conserved RX(R/K)R motifs, releasing the catalytic domain from inhibition and activating the protease. (**B**) Regulation by membrane trafficking complexes: Surface delivery and the activation of ADAM proteases are coordinated by accessory proteins/chaperones/co-factors. Tetraspanins (e.g., Tspan15) promote ADAM10 ER exit and induce an open extracellular conformation that enhances substrate access, as supported by recent cryo-EM structures. iRhom2 forms a stable complex with ADAM17, regulating its trafficking and preventing premature activation by interacting with its prodomain. (**C**) Intracellular domain (ICD) steric hindrance: The intracellular domains (ICDs) of certain substrates can modulate ADAM proteolysis by sterically inhibiting ADAM/substrate association. This includes ICD conformational changes (e.g., Eph/ephrin complexes) or interactions with calmodulin (e.g., L-selectin). (**D**) Autoinhibitory conformation: Structural studies of the ADAM10 ECD indicate that ADAM protease activity is tightly regulated by autoinhibitory intramolecular interactions between the metalloproteinase (MP) and cysteine-rich (C) domains that block substrate binding, which, in the case of ADAM10 at least, is relieved by interactions with Tspan15. Additional evidence suggests that activation also involves disulfide rearrangement within conserved thioredoxin-like CxxC motifs in ADAM10/17, which is mediated by protein disulfide isomerase (PDI). PDI interacts with the membrane-proximal region to reposition the extracellular domain, exposing the catalytic site. Redox changes further regulate PDI and ADAM activity. (**E**) Phosphorylation-dependent TIMP3 release: Surface ADAM proteases are maintained in an inactive state through TIMP3 binding. Phosphorylation at key intracellular residues (e.g., Thr719/735 in ADAM10/17) by MAPKs disrupts TIMP3 association, promoting the activation of the catalytic domain. Created with BioRender.com.

The activity of ADAM members is controlled by multiple mechanisms, including zymogen maturation, intracellular trafficking, conformational regulation, and specific cellular regulatory pathways (Figure 2).

### 3.1. Modulation by Accessory Proteins and Membrane Trafficking

The trafficking, localization, and maturation of ADAMs are an important part of their regulation and have been shown to be dependent on accessory or chaperone proteins, in particular tetraspanins (Tspans) and rhomboid-like proteins (iRhoms). TspanC8 proteins, named for the eight conserved cysteine residues within their extracellular region and including Tspans 5, 10, 14, 15, 17, and 33, are particularly important for the regulation of ADAM10 [28,29,30]. These proteins promote the transport of ADAM10 from the ER to the cell membrane and facilitate its inhibitory-domain removal. Studies using chimeric constructs revealed that the large extracellular loop of Tspan14 mediates its binding to ADAM10 and promotes trafficking and maturation. The membrane-proximal stalk, cysteine-rich, and disintegrin domains of ADAM10 are responsible for interacting with TspanC8s and are essential for its ER exit. Importantly, different TspanC8s impose distinct structural configurations on ADAM10, which in turn affect its substrate selectivity. Tspan15 uniquely promotes the cleavage of N-cadherin, while Tspan14 reduces the cleavage of the platelet collagen receptor GPVI [31]. Tspan15 was shown to co-localize with ADAM10 in a functional scissor complex, with Tspan15 stability dependent on the presence of ADAM10. Furthermore, antibodies targeting Tspan15 impaired the function of the ADAM10/Tspan15 complex, highlighting their interdependence [32]. The underlying mechanism was recently elucidated by a cryo-EM structure of ADAM10 in a complex with Tspan15 bound by an antibody Fab fragment, which suggests that the Tspan15-bound structure forms a more open conformation, enhancing activity against N-cadherin and potentially contributing to substrate specificity by repositioning the active site at a defined (~20 Å) distance from the plasma membrane [33]. How this role of activator combines with the trafficking of ADAMs to the plasma membrane is yet to be fully clarified.

The rhomboid-like proteins iRhom1 and iRhom2 are key regulators specifically controlling ADAM17 maturation and transport to the cell surface [34]. Like tetraspanins, iRhoms do not possess catalytic activity but instead facilitate the formation of proteolytic complexes with ADAM17. iRhom2 is highly expressed in immune cells and other cell types under inflammatory conditions [35] and is essential for ADAM17-dependent shedding of substrates like TNF-α. When iRhom2 is deficient, ADAM17 remains in the ER, as it is unable to cleave its substrates, leading to impaired inflammatory responses [35,36]. ADAM17 is essential for the stabilization of endogenous iRhom2, but not iRhom1, indicating that ADAM17 and iRhom2 form a functional complex in the ER, enabling ADAM17 maturation and trafficking, with both proteins mutually dependent for proper surface expression and activity [37]. Recent structural studies show that iRhom2 interacts with ADAM17 through both extracellular and transmembrane domain interactions. Interaction with the first transmembrane domain (TMD1) of iRhom2 is essential for the trafficking of ADAM17 from the ER to the Golgi and for maturation. The disruption of this interaction halts ADAM17 maturation, mimicking the phenotype of iRhom2 loss. Interactions also exist between the unique iRhom extracellular domain loop region between TMD1 and TMD2 via three distinct interfaces in the ADAM17 extracellular domain, with the most functionally important being an unexpected interaction with the prodomain. It is postulated that this allows the ADAM17 prodomain to remain transiently associated with iRhom2 following furin-mediated cleavage, suppressing premature activation until cytoplasmic cues trigger its release, enabling full protease activity [38].

The expression of iRhom2 is accordingly also important in cancer. It has been associated with the dysregulation of ADAM17-mediated erbB/EGF receptor signalling in Kras-mutant lung cancer via the phosphorylation of iRhom2, the recruitment of phospho-binding proteins, and the shedding of erbB ligands [39]. The importance of iRhom2 has also been revealed by pathogenic mutations in esophogeal cancer, where N-terminal mutations are associated with increased ADAM17- and protein-dependent EGFR signalling [40]. Thus, iRhoms are crucial for the regulation of ADAM17-mediated signalling and inflammation, representing potential therapeutic targets for inflammatory diseases.

### 3.2. Prodomain Cleavage and Activation

Most ADAMs are synthesized as inactive zymogens, requiring the removal or displacement of the prodomain for their activation. This generally occurs within the secretory pathway through cleavage by proprotein convertases (PCs) like furin, which target a conserved RX(R/K)R sequence motif [41,42]. The prodomain itself functions as a “cysteine switch”, a mechanism also occurring in matrix metalloproteinases whereby the interaction between a conserved cysteine residue and the zinc atom in the catalytic domain blocks the active site, and activation requires cleavage, reduction, or allosteric perturbation. This was corroborated by mutational studies showing the inhibition of ADAM10 activation upon the disruption of the PC cleavage site [43,44,45].

ADAM activity is also controlled by additional structural configuration and conformational dynamics. Xray crystallography of the structure of the full extracellular domain of ADAM10 revealed an autoinhibited state in which the cysteine-rich domain interacts with the metalloproteinase domain, preventing substrate access. The disintegrin and cysteine-rich domains form an elongated structure stabilized by disulfide bonds and hydrophobic interactions, ensuring structural integrity while maintaining enzymatic inactivity [19,20].

Another possible driver of conformational changes likely involves disulfide bond rearrangements between conserved thioredoxin CxxC motifs present in both ADAM10 and ADAM17 to expose the catalytic site. In support, the ADAM10 monocloncal antibody (mAb) 8C7 specifically binds to the active form of ADAM10, which is dependent on the intact CxxC motif near its binding epitope [46]. Disulfide bond switching between CxxC domains is driven by protein disulfide isomerase (PDI) activity [47] and is influenced by redox changes [48]. PDI was shown to directly interact with the membrane-proximal domain (MPD) of ADAM17 to induce the reorientation of the extracellular domains and thereby regulate shedding activity [49].

### 3.3. Dimerization, TIMPs, and Intracellular Signalling

ADAM activity is believed to be further regulated via dimerization, which depends on intracellular signalling. In the absence of MAPK stimulation, ADAM17 exists as dimers on the cell surface and is shown to favour binding and inhibition by the tissue inhibitor of metalloproteinase-3 (TIMP3) [50]. TIMPs (TIMPs 1–4) are natural inhibitors of metalloproteases; notably, TIMP3 potently inhibits both ADAM17 and ADAM10, while TIMP1 exhibits weaker inhibition of ADAM10 [51,52]. Upon the activation of ERK or p38 MAPK signalling, ADAM17 shifts to a monomeric form, resulting in TIMP3 release. Phosphorylation at Thr735 by the p38α MAP kinase enhances ADAM17 cleavage activity and reduces its association with TIMP3 [26,50]. Like ADAM17, ADAM10 also dimerizes, a process which is dependent on the transmembrane domain [22], and can undergo phosphorylation of the intracellular domain [53]. Interestingly, transient activation of ADAM17 has been described, which is not reliant on prodomain processing or TIMP3 dissociation, suggesting that there may be highly dynamic inhibition of the catalytic site [15]. The cytoplasmic domains of ADAMs 10 and 17 have been shown to be not required for activity but may rather play an inhibitory role via ER retention or via interactions with other proteins [15,54,55].

Conformational changes in the cytoplasmic domain of substrates have also been shown to play an important role in regulating ADAM-mediated proteolysis by modulating inhibitory interactions with the ADAM10 cytoplasmic domain. The intracellular domains (ICDs) of L-selectin and the angiotensin-converting enzyme interact with calmodulin to inhibit cleavage of their extracellular domains, and disruption of this interaction, such as through mutations in the L-selectin ICD, enhances ectodomain cleavage, thereby linking cytoplasmic domain conformation to proteolytic activation. Additionally, calmodulin binding to the proform of ADAM10 prevents its maturation [56]. While the mechanisms are not fully understood, intracellular steric hindrance of substrate interactions, which also extends to conformational changes in the ICDs of other substrates, may represent a broader mechanism of regulation, such as those observed in Eph receptor/ligand complexes, where phosphorylation and the ensuing conformational extension of the Eph cytoplasmic domain were shown to facilitate ADAM10-mediated shedding [55].

## 4. Major ADAM Family Members in Cancer

Given their crucial roles in proteolysis, cell adhesion, and signalling, the dysregulation of ADAM family members has been implicated in various pathological conditions. Among these, cancer has emerged as a major context in which ADAMs contribute to disease progression. The aberrant expression and activity of specific ADAMs can promote tumour growth, invasion, metastasis, and resistance to therapy. Published data show that several ADAMs, including ADAM8, ADAM9, ADAM10, ADAM12, and ADAM17, are overexpressed in tumours [9,57]. Multiple functions through which ADAMs may contribute to the initiation and advancement of cancer development have been proposed (Table 1), as described below.

### 4.1. ADAM8

ADAM8, an active protease first identified in macrophages and macrophage-like cell lines, was discovered through a cDNA library screen for genes upregulated in response to lipopolysaccharides (LPSs) [16]. ADAM8 sheds various chemokines, cytokines, and receptors, including the immunomodulatory low-affinity IgE receptor CD23, pro-TNF-α, CX3CL1, SCF, tumour necrosis factor receptor 1 (TNF-R1), and interleukin-1 receptor 2 (IL-1R2) [109]. Additionally, ADAM8 cleaves extracellular matrix (ECM) components such as collagen I, fibronectin, and periostin, which are important elements of the cancer stroma [58]. Its disintegrin domain enables interactions with integrins, particularly the β1-integrin subunit, facilitating downstream signalling pathways involving focal adhesion kinase (FAK), extracellular signal-regulated kinase 1/2 (ERK1/2), and protein kinase B (AKT/PKB) [63]. It is also known to shed the neuronal cell adhesion molecule close homologue of L1 (CHL1), promoting neurite outgrowth [63]. The upregulation of ADAM8 expression has been observed in endothelial cells during angiogenesis following tissue injury, underscoring its role in tissue repair and vascular remodelling [62].

ADAM8 has emerged as a significant player in various cancers, highlighting its potential as both a diagnostic and therapeutic target. In pancreatic ductal adenocarcinoma (PDAC), an immunohistochemical analysis of patient samples revealed ADAM8 expression in macrophages, natural killer (NK) cells, and particularly neutrophils. Notably, the quantity of ADAM8-positive neutrophils in post-capillary venules correlated with patient survival following surgery, indicating that it could be a useful diagnostic indicator [61]. In the context of breast cancer, ADAM8 was found to be widely expressed and associated with a poor prognosis. Immunohistochemistry (IHC) and a breast cancer cell line microarray revealed that ADAM8 was present in approximately one-third of breast cancers, with high levels linked to adverse outcomes in hormone receptor-positive/HER2-negative subtypes [64,65]. Two novel truncated ADAM8 isoforms, Δ18a and Δ14′, were identified in lung cancer cells but not in normal tissues. These isoforms encode shortened proteins truncated at the transmembrane domain or within the cysteine-rich domain, respectively. Functionally, Δ18a enhanced invasive activity in vitro, while Δ14′ promoted osteoclast formation by increasing IL-6 and IL-8 secretion and contributed to bone metastasis by increasing tumour burden and osteolytic activity in vivo [60]. In prostate cancer, ADAM8 expression was significantly associated with higher tumour stages, nodal involvement, and Gleason scores, although its value in predicting relapse-free survival was limited [59]. ADAM8 suppression significantly reduced the proliferation, migration, and invasiveness of clear cell renal cell carcinoma (ccRCC) cells. Moreover, the combined application of ADAM8 knockdown and PD-1 blockade effectively inhibited tumorigenesis in ccRCC models and lead to the prolonged survival of mice [110].

Recent studies investigating glioblastoma have shown that chemotherapy with temozolomide (TMZ) induces an upregulation of proteases such as ADAM8, ADAM10, and ADAM17, which are likely involved in the cleavage of immunomodulatory molecules like PD-L1. ADAM8 was identified as a key mediator of soluble PD-L1 (sPD-L1) generation in glioblastoma cells, with knockout models confirming that sPD-L1 release is largely dependent on ADAM8. Although surface PD-L1 reduction could appear beneficial, the accumulation of sPD-L1 in the tumour microenvironment may still suppress T cell function at a distance, thereby contributing to immune evasion [67].

### 4.2. ADAM9

ADAM9, also known as meltrin-γ, was initially identified in breast carcinoma. It is broadly expressed in diverse human tissues and is notably upregulated in pathological states. It is present in various cell types, including but not limited to monocytes, macrophages, neutrophils, keratinocytes, epithelial cells, and fibroblasts, and is found in a wide range of tissues such as the lungs, colon, kidneys, vascular smooth muscle, nervous system, reproductive system, and secretory organs [111]. Initially discovered as a facilitator of muscle cell fusion during development, ADAM9 is implicated in myogenesis and myotube formation, including the cardiac myocardium. However, ADAM9 knockout mice develop normally, remain viable and fertile, and exhibit no abnormalities, indicating that other ADAMs may compensate for its absence during development [76]. ADAM9 also plays a role in the formation of the endocardial cushion during embryogenesis [70] and is involved in shedding the interleukin-11 receptor [74].

In PDAC tumour cells, ADAM9 expression is elevated and correlates with poor tumour grading and vascular invasion [73]. The loss of ADAM9 increases the interaction between KRAS and plasminogen activator inhibitor 1 (PAI-1), which functions as a selective autophagy receptor with light chain 3 (LC3), leading to lysosomal degradation of KRAS. Inhibiting ADAM9 with a small-molecule inhibitor restricts disease progression in spontaneous models and, when combined with gemcitabine, induces significant regression of patient-derived tumours [71]. In colorectal cancer cells, ADAM9-driven cleavage of ephrin-B was reported to trigger the activation of the Akt pathway, stimulating downstream Wnt and mTOR signalling cascades and enhancing cellular migration and invasion [112]. In renal cancer, ADAM9 is significantly overexpressed compared to adjacent normal tissue, and its expression is associated with a higher tumour grade, positive nodal status, and distant metastasis and correlates with reduced patient survival in univariate analysis [69]. The knockdown of ADAM9 in MDA-MB-231 breast cancer cells inhibits tumour cell invasion in vitro [72]. Immunohistochemical analysis reveals significantly higher ADAM9 expression in oral squamous cell carcinoma (OSCC) tissues compared to normal tissues [75], and it is also markedly upregulated in prostate cancer at both mRNA and protein levels [69]. Thus, ADAM9 is upregulated in various tumour types, suggesting its significant potential as a therapeutic target.

### 4.3. ADAM12

ADAM12 plays a central role in the ectodomain shedding, cleaving, and releasing of the extracellular portions of proteins such as pro-heparin-binding EGF (proHB-EGF), insulin-like growth factor-binding proteins IGFBP-3 and IGFBP-5, oxytocinase, GPNMB, and basigin [113,114]. It exists in two isoforms produced by alternative splicing: a transmembrane form (ADAM12-L) and a secreted form (ADAM12-S), with the latter consisting only of the extracellular region. It is frequently overexpressed in various malignancies and has context-specific roles in tumour progression [115].

Several experimental studies have investigated ADAM12’s role using both in vitro and in vivo models. For instance, genome-wide DNA methylation profiling and expression analysis reveal that ADAM12 is hypomethylated and overexpressed in TNBC and in adjacent tissue, which may suggest early epigenetic dysregulation. Functional experiments confirmed that ADAM12 promotes proliferation, migration, and doxorubicin resistance in TNBC cells, and its low methylation correlates with poor patient survival [80]. Further studies in breast cancer identified ADAM12 as a regulator of angiogenesis. Co-culture assays showed that endothelial cells exposed to ADAM12-expressing tumour cells exhibited increased recruitment and capillary tube formation. This was associated with the upregulation of VEGF and MMP-9 and the suppression of anti-angiogenic regulators like THBS1 and TIMP-2, suggesting a pro-angiogenic shift mediated by ADAM12 [82]. Additionally, in claudin-low breast cancer cells, ADAM12 was shown to maintain the cancer stem cell (CSC) phenotype through the modulation of EGFR signalling [78]. Another study demonstrates that under hypoxic conditions, HIF-dependent upregulation of ADAM12 promotes breast cancer metastasis by enhancing HB-EGF shedding and activating EGFR–FAK signalling. Using dynamic motility assays and gene knockdown models, the authors show that ADAM12 is essential for hypoxia-induced cell migration and invasion, acting in parallel with other HIF-regulated pathways [83].

In hepatocellular carcinoma, clinical dataset analyses revealed a strong correlation between high ADAM12 expression and an advanced tumour stage [77]. Bioinformatics analyses in colon adenocarcinoma revealed that ADAM12 is overexpressed and linked to a poor prognosis. Its expression correlates with immune cell infiltration, immunomodulators, and chemokines, suggesting a role in extracellular matrix remodelling and immune microenvironment regulation [79]. Recently, irradiation-induced upregulation of ADAM12 was observed in mouse and human colon cancer cells, as well as rectal cancer patient tumour tissues. While ADAM12 knockdown did not alter intrinsic radiosensitivity in cell culture models, in vivo experiments using ADAM12-deficient CT26 cells revealed significantly delayed tumour growth and improved survival following irradiation, implicating ADAM12 in modulating the tumour microenvironment (TME) rather than direct cell-autonomous effects. Interestingly, unlike in the breast cancer studies above, endothelial cells were more prominent in ADAM12-depleted tumours, and in vitro studies showed that there was increased endothelial cell tube formation in response to conditioned media from irradiated ADAM12 knockout cells, suggesting enhanced vascular remodelling [81].

Together, these studies highlight the diverse roles of ADAM12 in cancer progression, ranging from its involvement in angiogenesis, metastasis, and therapy resistance to its potential as a prognostic biomarker, thereby warranting further investigation into its clinical relevance across tumour types.

### 4.4. ADAM10

ADAM10 was initially identified as a protease that degrades myelin basic protein in the bovine brain, producing a 748-amino-acid protein with a molecular mass of 100 kDa in its proform and 60 kDa in its active, mature form [89]. ADAM10 plays a crucial role in several developmental and physiological processes, such as the formation of somites/tissue boundaries and the neural and vascular systems, and in mediating inflammatory responses. Its substrates include ligands and/or receptors from the Notch, EGF/erbB, and Eph receptor families as well as various cytokines (TNFα), cytokine receptors (IL-1R, IL-6R, and TNF-RI), chemokines, and adhesion molecules (L-selectin and cadherins) [42,86,89]. It is essential for development, and ADAM10 knockout mice phenocopy Notch knockout mice, highlighting Notch as a key substrate [91]. As in the case of ADAM8, recent studies have shown that both ADAM10 and ADAM17 may also cleave PD-L1, with their expression and activation correlating with soluble PD-L1 levels, a mechanism that can suppress anti-tumour immunity by promoting apoptosis in CD8+ T cells, particularly in triple-negative breast cancer (TNBC) [11,94,116].

In TNBC, the knockdown of ADAM10 or treatment with the ADAM10-selective inhibitor GI254023X significantly reduced cell migration. Moreover, high ADAM10 mRNA levels have been linked to poor outcomes in basal subtype breast cancer patients [93]. ADAM10 is implicated in promoting migration, invasion, cell growth, and cell cycle progression and preventing apoptosis in TNBC cells through Notch pathway activation [88]. Similarly, ADAM10 overexpression in prostate cancer xenografts in mice facilitated tumour growth and metastasis, particularly in the lungs and lymph nodes, and was associated with elevated angiogenic markers CD31 and VEGF [87].

In non-small cell lung cancer (NSCLC), ADAM10 is overexpressed in malignant tissues and metastatic sites compared to normal tissues. The knockdown of ADAM10 significantly inhibited the migration and invasion of NSCLC cells, suggesting a role in promoting metastasis [90]. Moreover, ADAM10 sheddase activity was reported to be notably higher in aggressive NSCLC tumours and in exosomes from the blood of NSCLC patients, suggesting its potential as a significant biomarker, despite inconsistent protein expression levels in primary tumours [96].

In osteosarcoma, ADAM10 enhances cell growth, migration, and invasion. Silencing ADAM10 in these cells leads to the inactivation of the E-cadherin/β-catenin signalling pathway, which is characterized by increased E-cadherin levels, reduced nuclear β-catenin translocation, and decreased MMP-9, Cyclin D1, and c-Myc levels [97].

ADAM10 is also overexpressed in pancreatic ductal adenocarcinoma (PDAC) compared to normal pancreatic tissues, contributing to cancer cell invasiveness and metastasis, although it does not affect cell viability [89]. ADAM10-mediated cleavage of the Eph receptor ligand ephrin-B2 has been linked to fibrosis in pancreatic cancer, with radiation therapy shown to enhance ADAM10 expression on tumour cells and its cleavage of ephrin-B2 from stromal fibroblasts, thereby promoting tumour survival and fibrosis. High ADAM10 expression in pancreatic tumours correlates with a poor prognosis, indicating a role in tumorigenesis [92]. ADAM10 is similarly elevated in other GI cancers, including gastric cancer, where higher ADAM10 levels are also linked to invasion, metastasis, and a poor prognosis [95]. In colon cancer, high levels of ADAM10, particularly a tumour-selective high-molecular-weight (unprocessed) form, are associated with advanced disease [46,85]. Autoantibodies recognizing the HMW form of ADAM10 were detected in patient sera and correlated with a better prognosis, highlighting its potential as a therapeutic target [85]. In support, treatment with the anti-ADAM10 mAb 8C7 inhibited the growth of colon xenografts in mice [46].

### 4.5. ADAM17

ADAM17, originally recognized for its role in cleaving the proform of tumour necrosis factor α (TNF-α) and hence also named TNF α-converting enzyme (TACE), plays a crucial role in development, inflammation, and immunity. Other substrates include certain EGFR ligands, including amphiregulin, epiregulin, heparin-binding EGF-like growth factor, and transforming growth factor α (TGF-α) [98,99]. Like its close relative ADAM10, ADAM17 is essential for normal development, and ADAM17 KO mice resemble mice lacking TGF-α [100].

In triple-negative breast cancer (TNBC), ADAM17 promotes malignancy by driving proliferation, invasion, and angiogenesis through the activation of the EGFR-PI3K-AKT signalling pathway. This activation is facilitated by increased secretion and expression of TGF-α and VEGF, which can be mitigated by ADAM17 siRNA [117]. Similarly, ADAM17 activates the EGFR-PI3K-AKT pathway in glioma, resulting in enhanced proliferation, invasion, and angiogenesis, and inhibition of ADAM17, which significantly reduce tumour growth in mice [108]. ADAM17-mediated cleavage of TGF-α also plays a role in prostate cancer, where the overexpression of ADAM17 correlates with invasive characteristics, the activation of the EGFR-MEK-ERK pathway, and increased MMP-2 and MMP-9 levels. Accordingly, the blockage of TGF-α shedding was shown to prevent cell invasion [107].

ADAM17 also plays a critical role in KRas-driven malignancies, such as subtypes of colorectal cancer (CRC), pancreatic cancer, and lung adenocarcinoma (LAC) [104]. One mechanism is by inducing IL-6 trans-signalling by shedding sIL-6R, a process particularly important in KRas-mutant cancers, where it promotes tumour growth and progression, contributing to resistance against EGFR-targeted therapies. In KRasG12D-driven LAC, genetic disruption or inhibition of ADAM17 significantly reduces tumour growth by impairing cell proliferation. This effect was linked to p38 MAPK-mediated phosphorylation of ADAM17, the release of the soluble IL-6 receptor (sIL-6R) and the activation of IL-6 trans-signalling via the ERK1/2 pathway. Notably, ADAM17’s pro-tumorigenic effect in LAC is independent of immune cell involvement, with phosphorylated ADAM17 predominantly localized to epithelial cells [106]. It also plays a role in non-small cell lung carcinoma (NSCLC), as silencing ADAM17 expression via siRNA can inhibit tumour cell proliferation and invasion in vitro, as well as tumour growth in vivo [102].

In CRC, ADAM17-mediated IL-6 trans-signalling and subsequent STAT3 activation drive tumour growth, as well as inflammation and immune evasion. They also mediate EGFR signalling in myeloid cells, triggering IL-6 production, which acts on intestinal epithelial cells to promote tumorigenesis. Blocking this pathway using sgp130Fc disrupts IL-6 trans-signalling, offering a potential therapeutic strategy for CRC treatment [105]. Additionally, shikonin (SKN), a natural antitumor agent, suppresses ADAM17 expression through ROS-mediated translational inhibition, which reduces both constitutive and IL-6-induced STAT3 activation, thereby suppressing colon cancer cell proliferation. The reversal of this effect by the ROS scavenger NAC may support a role for ROS in regulating ADAM17 expression as well as activation (Section 3) [106]. Interestingly, ADAM17 expression is detected on circulating exosomes in blood from CRC patients and has been associated with metastasis, suggesting its utility as a biomarker for metastatic potential [101]. An investigation in lab-based models showed that exosome-associated ADAM17 compromises endothelial cell adhesion by cleaving VE-cadherin, thereby increasing vascular permeability and enabling cancer cell intravasation. In mice, exosomal ADAM17 mediated the formation of a pre-metastatic niche by inducing vascular leakage and promoted CRC metastasis [101]. The multifaceted roles of ADAM17 in CRC indicate significant potential for therapeutic targeting.

In pancreatic ductal adenocarcinoma (PDAC), ADAM17/TACE mRNA expression is upregulated in tumour-derived tissues and cell lines, and siRNA silencing of ADAM17 significantly reduced cancer cell invasion in lab-based models, although proliferation remained unaffected [118]. ADAM17 enhances ErbB signalling in PDAC by cleaving precursor ligands into soluble active growth factors. The inhibition of ADAM17 using the mAb A9(B8) delayed tumour development in the Pdx1Cre; KrasG12D; Trp53fl/+ (KPC) model [119]. Ardito et al. further identified that oncogenic KRAS elevates both the expression and activation of EGFR, with its activation being contingent on the ligand-shedding function of ADAM17. Experimental models showed that genetic disruption or inhibition of EGFR or ADAM17effectively blocked KRAS-induced PDAC tumour growth in vivo [120]. Moreover, KRAS mutations enhance ADAM17-mediated cleavage of the erbB3/4 ligand neuregulin 1 (NRG1), further activating oncogenic pathways via ErbB receptor signalling [121].

Interestingly, ADAM17 is also upregulated in pancreatic tissues during pancreatitis, a precursor to pancreatic ductal adenocarcinoma (PDAC). In acute pancreatitis (AP) models, both genetic and therapeutic inhibition of ADAM17 improved outcomes. In a chronic pancreatitis (CP) model, genetic suppression resulted in reduced pancreatic fibrosis. ADAM17 inhibition suppressed IL-6 trans-signalling/STAT3 activation, decreasing inflammation and necrosis. Clinically, the ADAM17/IL-6/STAT3 pathway is elevated in pancreatitis patients, linking ADAM17 to both inflammation and potential PDAC development [122].

## 5. Recent Advances to Target ADAMs

Several strategies exist for inhibiting ADAM protease activity, including the use of low-molecular-weight synthetic inhibitors, the ADAM prodomain (either purified or synthetic), modified TIMPs, and monoclonal antibodies [123] (Table 2).

### 5.1. ADAM8

Targeting ADAM8 with the peptide mimetic has been shown to enhance survival in pancreatic cancer (KPC) mouse models by reducing tumour volume, limiting metastasis, and decreasing tumour cell invasion while preserving acinar structures [124]. BK-1361 is a cyclic peptide mimicking the RLSKDK motif in the disintegrin domain of mouse ADAM8. This modification hinders the multimerization of ADAM8 by binding directly to the disintegrin domain, preventing its interaction with β1 integrin on the cell surface [124,147]. In TNBC mouse models, an ectodomain antibody against ADAM8 was reported to decrease primary tumour growth, inhibit metastasis formation, and reduce the size of existing metastases, suggesting a new therapeutic approach for TNBC [65]. Furthermore, a panel of highly specific monoclonal antibodies against ADAM8, identified through hybridoma screening, has shown significant anticancer activity in TNBC models, with two lead antibodies, ADP2 and ADP13, reducing tumour growth and metastasis and improving survival in mice [148]. Additionally, propofol, a common anesthetic, has been studied for its effects on cancer cells, particularly its impact on ADAM8. Low doses of propofol have been shown to inhibit pancreatic cancer cell proliferation, migration, and invasion by downregulating ADAM8 expression, an effect mediated by the suppression of specificity protein 1 (SP1), which regulates ADAM8 transcription. These findings link propofol’s anticancer effects to reduced ADAM8 mRNA and protein levels, with SP1 playing a key role in this mechanism [125].

### 5.2. ADAM9

ADAM9 expression can be inhibited by tyrosine kinase inhibitors such as sorafenib and regorafenib [127,128]. Sorafenib, a multi-receptor tyrosine kinase inhibitor used clinically for hepatocellular carcinoma (HCC), has been reported to decrease ADAM9 mRNA and protein levels. This resulted in increased expression of membrane-bound MICA (MHC Class I polypeptide-related sequence A), a cancer cell ligand that triggers natural killer cell attack, and it is an ADAM9 substrate. Reducing ADAM9 levels lead to increased MICA expression on the cell surface and enhanced natural killer cell activity against HCC [128]. Similarly, regorafenib, another multi-kinase inhibitor, lowered ADAM9 and additionally ADAM10 levels in sorafenib-resistant HCC cases, contributing to the accumulation of MICA on HCC cell membranes and the dual inhibitory effect partially explaining its clinical advantage over sorafenib [127]. Leukotriene receptor antagonists, such as pranlukast and montelukast, have been found to inhibit ADAM9 activity in vitro and elevate mMICA levels in HCC cells [126]. Natural flavonoids also influence ADAM9 expression and function. Fisetin was shown to reduce ADAM9 expression, and the migration and invasion of GBM8401 glioma cells were inhibited by enhancing sustained ERK1/2 phosphorylation [129], and similarly, this was observed in renal cell carcinoma [130], where it was counteracted by an ERK inhibitor. Galangin and Licochalcone A have similar effects also involving ERK pathway activation in glioma cells [149]. In a different approach, targeting ADAM9 with the novel antibody–drug conjugate IMGC936 has shown preclinical efficacy by causing cytotoxicity in ADAM9-positive tumour cell lines and potent antitumor activity in xenograft models [131].

### 5.3. ADAM12

Loechel et al. found that recombinant TIMP-3 effectively inhibits recombinant ADAM12 in vitro, with a stronger effect observed at higher ratios, suggesting its role as a regulator of ADAM12 activity, although this has not verified in vivo [44]. Asakura et al. tested KB-R7785, a small molecule inhibitor of ADAM12, in mice with induced cardiac hypertrophy. KB-R7785, which binds directly to ADAM12, prevents the shedding of heparin-binding epidermal growth factor (HB-EGF). This inhibition blocks the subsequent activation of the EGF receptor (EGFR) and the hypertrophic response. Treatment with KB-R7785 (100 mg/kg/day) resulted in a significant decrease in heart weight and the heart-to-body weight ratio, indicating reduced hypertrophy, while heart rate, blood pressure, and body weight remained unchanged [132].

### 5.4. ADAMs 10 and 17

The ability to precisely inhibit ADAM metalloprotease activity remains a challenge due to similarities of the catalytic site with those in MMPs. Most studies initially focused on broad-spectrum small-molecule hydroxamate inhibitors; following their development for MMPs, clinical trials discontinued due to a lack of specificity that led to toxicity. GI254023X is a small-molecule ADAM10 inhibitor which was shown to inhibit the cleavage of the Il-6 receptor, CX3CL1, and CXCL16 in cell-based tests [9,117,123,133]. However, GI254023X also inhibits MMP2 and MMP9, as well as ADAM17, albeit with 100-fold lower potency compared to its inhibition of ADAM10 [4,134,135]. TAPI-1 and TAPI-2 are strong inhibitors of ADAM-17 (TACE), and can effectively prevent the shedding of cytokine receptors such as IL6R. However, they may also influence matrix metalloproteinases (MMPs) [150,151]. CID 3117694 is a first-in-class, non-zinc-binding, time-dependent exosite inhibitor of ADAM10 that selectively blocks the cleavage of glycosylated substrates such as CXCL16 by competing with O-glycosylated residues for exosite binding. It demonstrates substrate-selective inhibition in vitro and exhibits significant efficacy in a collagen-induced arthritis (CIA) mouse model, reducing disease scores, inflammation, and cytokine levels without observed toxicity [152,153]. Rapamycin and triptolide are natural compounds that inhibit ADAM10; rapamycin blocks ADAM10 maturation, while triptolide reduces ADAM10 expression and promotes its degradation, though their exact mechanisms remain unclear [135]. Selective hydroxamate-based ADAM10 inhibitors (LT4 and MN8) reduce Hodgkin lymphoma cell growth, inhibit CD30 and TNFα shedding, and enhance the anti-tumour activity of brentuximab vedotin in 3D culture models that mimic the lymph node microenvironment, highlighting their potential for combinatory therapy development [154].

INCB3619 is a small-molecule inhibitor of both ADAM10 and ADAM17 which demonstrated notable efficacy in preclinical studies. Initial research suggested that INCB3619 could obstruct the release of the HER3 ligand heregulin in non-small cell lung cancer (NSCLC) models, which in turn enhanced the effectiveness of gefitinib. Additionally, it was observed to increase apoptosis and reduce the response threshold to paclitaxel in NSCLC cells [137]. While it offers improved selectivity for ADAM proteases over MMPs, its dual targeting action, as well as its retained low affinity for certain MMPs, does not rule out possible off-target effects [136]. The related, second-generation compound INCB7839 showed synergistic effects in inhibiting growth when combined with the dual EGFR/HER2 inhibitor GW2974 in MCF-7 breast cancer cells. Clinical evaluations began in 2007 for HER2-positive breast cancer, where INCB7839 was found to be generally well tolerated. Further Phase I/II trials involving INCB7839 and Herceptin indicated improved responses in patients expressing the p95 truncated form of HER2, who are resistant to Herceptin alone [138]. The next generation of INCB8765 is an ADAM10-specific inhibitor that blocks EGF ligand processing in non-small cell lung cancer (NSCLC) cells in vitro [137]. While it is more selective for ADAM10 over ADAM17 and MMPs, it only shows a 20-fold difference in IC50 values between ADAM10 and ADAM17, which does not preclude broader ADAM inhibition, and no clinical data are available [136].

Various antibodies targeting ADAM10 and ADAM17 have also been developed, particularly for ADAM17. The D1(A12) antibody binds to both catalytic and non-catalytic domains of ADAM17, demonstrating significant potency in biochemical assays. This antibody was reported to inhibit the shedding of several ADAM17 substrates, including TNFα, TGFα, AREG, and HB-EGF. In vivo studies revealed that D1(A12), a cross-domain antibody that binds to both the M and D+C domains of ADAM17, was effective in reducing the tumour burden in mice with IGROV1-Luc tumours [139,140]. Another antibody, MEDI3622, specifically targets the metalloprotease domain of ADAM17. MEDI3622 has shown remarkable efficacy in inhibiting tumour growth in CRC PDX models, including those with KRAS mutations. This treatment prolonged the survival of mice with tumour xenografts and was also found to influence Notch signalling and oncogenic progenitor cells [141]. It has also shown significant inhibition of amphiregulin shedding in NSCLC cells and demonstrated dose-dependent inhibition of tumour growth in oesophageal cancer models. MEDI3622 also enhanced NK cell activity when used in conjunction with trastuzumab or rituximab [142,143,144]. Similarly, the antibody D8P1C1 binds to and inhibits the M domain of ADAM17 and showed efficacy in a range of cell lines in vitro and in breast and ovarian xenografts in vivo [155]. Alternatively, the A300E antibody targets the membrane-proximal cysteine-rich region of ADAM17 and has been developed as a single-chain variable fragment (scFv) fused to a CD3-specific scFv to form a bispecific T-cell engager (A300E-BiTE). This construct has been shown to lyse prostate cancer cells in co-culture with peripheral blood mononuclear cells and selectively kill breast cancer cells when conjugated with doxorubicin or Pseudomonas exotoxin A [145,146].

Fewer antibodies have been developed thus far against ADAM10. The mAb 8C7 binds to the non-catalytic C domain of ADAM10 and preferentially binds to an active conformation of ADAM10 [20,46]. 8C7 blocked ephrin shedding and cell sorting in vitro [156], as well as inhibited tumour growth in mice with human colon cancer xenografts, associated with decreases in notch and Eph receptor function and vessel formation [46]. Similar results were seen with an analogous human antibody recognizing the same domain and preferentially targeting active ADAM10 [157]. The tumour selectivity of 8C7 was used as the basis for the subsequent development of 8C7 antibody–drug conjugates (ADCs), which significantly increase anti-tumour efficacy in colon and GBM xenograft models [158].

## 6. Conclusions, Challenges, and Future Directions

ADAMs are commonly over-expressed in a range of cancers, where they play essential roles in tumour progression, metastasis, and immune modulation through the proteolytic cleavage of growth factors, cytokines, and adhesion molecules. These multifaceted functions make ADAMs highly attractive therapeutic targets, although their targeting presents various challenges. One such challenge is the functional redundancy among ADAM family members, with several ADAMs sharing overlapping substrates and functions, which may allow cancer cells to bypass the inhibition of a specific ADAM and sustain tumour progression. This redundancy complicates therapeutic strategies, as selective inhibition of a single ADAM may not achieve lasting tumour control. Simultaneous inhibition of multiple ADAMs may increase efficacy but also raises the risk of greater systemic toxicity. Identifying and inhibiting dominant family members in different tumour types could help address this issue.

ADAM expression in normal tissues also presents a significant challenge, potentially leading to off-tumour effects and toxicity. Given their critical roles in tissue regeneration, immune modulation, and neuroprotection, the systemic targeting of ADAMs risks impairing essential physiological processes. To minimize such adverse effects, therapeutic strategies must prioritize the selective targeting of tumour cells over normal tissues, such as the targeting of tumour-specific, active forms. One promising approach involves local delivery methods, such as nanocarrier-based drug systems or antibody–drug conjugates, which can restrict inhibitor activity to the tumour microenvironment and preserve normal tissue function.

Additionally, the structural similarity of ADAM catalytic sites to matrix metalloproteinases (MMPs) adds complexity to the development of selective catalytic inhibitors that avoid off-target effects. Attempts to inhibit metalloproteases in cancer have been limited by poor specificity and adverse effects, and even later generations of more specific small-molecule ADAM inhibitors retain some cross-reactivity with MMPs. This highlights the need for novel approaches such as the allosteric modulation or targeting of non-catalytic functional domains specific to ADAMs. Furthermore, the regulation of ADAM activity is influenced by post-translational modifications, protein interactions, and the tumour microenvironment, factors that can modulate ADAM function. An emerging area of interest lies in disrupting the interactions between ADAMs and their regulatory cofactors, such as iRhoms and tetraspanins, which are critical for their maturation, trafficking, and substrate selectivity. Targeting these may offer a more refined approach to modulate ADAM function with greater context specificity.

Thus, despite numerous challenges, new and advanced strategies are being explored, such as optimized small-molecule inhibitors, monoclonal antibodies, antibody–drug conjugates, and combination therapies, to improve specificity and efficacy. Since ADAMs contribute to several physiological processes with protective or regenerative roles, a comprehensive understanding of ADAM expression, regulation, and function will be crucial to unlocking their full therapeutic potential in order to achieve both safety and efficacy in controlling aggressive tumour growth.

## Figures and Tables

**Table 1:** Expression of ADAMs in cancers, their functional roles, substrates, and association with tumour progression and patient prognoses.

ADAM	Cancer Type(s)	Key Functions in Cancer	Substrates	Prognosis	References
ADAM8	Pancreatic, Breast, Lung, Prostate, and GBM	Tumour growth, invasion, and metastasis	CD23, TNF-R1, IL-1R2 collagen I, fibronectin, periostin, KL1, TNFα, APP, CHL1, and PD-L1	Poor prognosis	[58,59,60,61,62,63,64,65,66,67]
ADAM9	Pancreatic, Renal, Breast, and Oral	Tumour cell invasion and vascularization	HB-EGF and the interleukin-11 receptor	Poor prognosis	[68,69,70,71,72,73,74,75,76]
ADAM12	Colon, Breast, and Liver	Migration, invasion, angiogenesis, and stem cell phenotype	EGFR	Poor prognosis and therapy resistance	[77,78,79,80,81,82,83,84]
ADAM10	Breast, Osteosarcoma, Pancreatic, and Lung	Cell migration, invasion, metastasis, fibrosis, and immune evasion	Notch, Eph/ephrins, TNF, CX3CL, CXCL16, L1-CAM, N-cadherin, and E-cadherin	Poor prognosis, with overexpression linked to metastasis	[11,42,46,85,86,87,88,89,90,91,92,93,94,95,96,97]
ADAM17	Pancreatic, Breast, Prostate, Colon, Lung, and especially K-Ras mutant tumours	Proliferation, invasion, and angiogenesis via EGFR activation	TNF-α, NRG1, Amphiregulin, Epiregulin, Heparin-binding EGF, and TGF-α	Poor prognosis linked to therapy resistance	[98,99,100,101,102,103,104,105,106,107,108]

**Table 2:** Small-molecule inhibitors, antibodies, and ADCs targeting ADAMs along with their clinical trial status.

Target	Drug	Type	Mechanism of Action (MOA)	Clinical Status	Clinicaltrials.gov Identifier, Reference
ADAM8	BK-1361	Small-MW inhibitor	Suppresses multimerization of ADAM8	Preclinical	[124]
ADAM8	Propofol	Small-MW inhibitor	Inhibition of specificity protein 1 (SP1), a key regulator of ADAM8 gene expression	Preclinical	[125]
ADAM9 (indirect)	Sorafenib and Regorafenib	Tyrosine kinase inhibitor	Reduces ADAM9/10 expression and MICA shedding, boosting natural killer cell activity	Preclinical	[126,127,128]
ADAM9	Fisetin	Natural flavonoid	Increasing ERK1/2 activation through phosphorylation	Preclinical	[129,130]
ADAM9	IMGC936	Antibody–drug conjugate	Delivers a cytotoxic DM21-maytansinoid payload to tumour cells, inducing cell death	Phase 1/2	[131] NCT04622774
ADAM12	TIMP3	Small-MW inhibitor	Blocking the substrate-binding site in the cysteine-rich domain	Preclinical	[44]
ADAM12	KB-R7785	Small-MW inhibitor	Binds the ADAM12 active site, blocks the release of HB-EGF, and activates EGFR	Preclinical	[132]
ADAM10, ADAM17	GI254023X	Small-MW inhibitor	Prevents the shedding of the IL-6 receptor	Preclinical	[4,9,117,123,133,134,135]
ADAM10	INCB8765	Small-MW inhibitor	Blocks EGF ligand processing (not selective)	Preclinical	[136,137]
ADAM10, ADAM17	INCB3619	Small-MW inhibitor	Prevents the secretion of the HER3 ligand neuregulin	Preclinical	[137]
ADAM10, ADAM17	INCB7839	Small-MW inhibitor	EGFR/HER2 inhibitor	Phase 1/2	[138] NCT02141451 NCT04295759
ADAM10	8C7	Antibody and ADCs	Targets active ADAM10 and inhibits the cleavage of membrane-bound ligands, Eph, and notch signalling	Preclinical	[20,46]
ADAM17	D1(A12)	Antibody	Attaches to both the M and D+C regions and blocks the release of various ADAM17 substrates	Preclinical	[139,140]
ADAM17	MEDI3622	Antibody	Binds the ADAM17 MP domain and inhibits amphiregulin shedding	Preclinical	[141,142,143,144]
ADAM17	A300E	Antibody	Binds the ADAM17 C domain; bispecific anti-CD3 promotes anticancer T cell activity	Preclinical	[145,146]
